# A Giant Ovarian Cyst Masquerading as Pregnancy: A Case Report

**DOI:** 10.7759/cureus.90319

**Published:** 2025-08-17

**Authors:** Fariha Sabeen

**Affiliations:** 1 Anatomy, Rajendra Institute of Medical Sciences, Ranchi, IND

**Keywords:** cystectomy, followed by abdominal pain with distension, giant ovarian cyst, menorrhagia, ovarian cystectomy, young female with abdominal pain

## Abstract

A 30-year-old woman presented with abdominal pain, amenorrhea, and a significantly enlarged abdomen. Initially, she believed she was pregnant due to the cessation of her menstrual cycles and gradual abdominal growth over several months. However, an ultrasound examination revealed a massive ovarian cyst, rather than a pregnancy. Due to the patient's extremely late presentation, which resulted in a giant ovarian cyst, a laparotomy was necessitated over laparoscopy, as the sheer size and complexity of the case demanded an open surgical approach to ensure safe and effective management. Despite the challenges posed by the case, the healthy ovary was successfully preserved, pertaining to the young age of the patient. This case underscores the importance of regular medical check-ups and timely ultrasounds for early detection and management of ovarian cysts. The absence of distinctive symptoms in this case made accurate preoperative diagnosis challenging, emphasizing the need for awareness and prompt evaluation to prevent complications. Early diagnosis and intervention are crucial in managing ovarian cysts and preventing potential complications, highlighting the significance of timely medical evaluation.

## Introduction

Giant ovarian cysts (GOCs), which are described in the literature as measuring more than 10 cm in size in their largest diameter, are rare in occurrence, particularly with the advent of advanced imaging modalities and routine physical examinations [1]. The ovaries, paired intra-peritoneal endocrine organs, play a crucial role in reproduction and hormone production. As a result of ovulation, a fluid-filled sac known as an ovarian cyst can form on one or both ovaries. Ovarian cysts are not uncommon, with 20% of women worldwide developing at least one pelvic mass in their lifetime. Various subcategories have been identified, characterizing more than 30 types of ovarian masses, and management is determined by the characteristics of the lesion, the patient's age, and risk factors for malignancy [2].

Most women of reproductive age develop small cysts each month. Simple, smooth ovarian cysts, smaller than 3 cm and apparently filled with water, are considered normal [3]. Large cysts that cause problems occur in about 8% of women before menopause. Ovarian cysts are present in about 16% of women after menopause, and have a higher risk of being cancerous than in younger women [4]. Simple cysts are not associated with an increased risk of ovarian cancer, whereas complex cysts or solid masses are associated with a significantly increased risk of ovarian cancer [5].

Benign ovarian cysts are common in asymptomatic premenarchal girls and are found in approximately 68% of the ovaries of girls aged two to 12 years and in 84% of the ovaries of girls aged 0 to two years. Most of them are smaller than 9 mm, while about 10-20% are larger macrocysts. While the smaller cysts mostly disappear within six months, the larger ones appear to be more persistent [6].

Abnormalities, such as cysts, can have significant effects on women's health. A thorough understanding of the various presentations of an ovarian cyst is vital for clinicians to accurately diagnose and manage them, including giant ovarian cysts. Ovarian cysts are generally asymptomatic at early stages, causing symptoms only after reaching enormous dimensions, and consequently, they are often diagnosed late [7]. The clinical symptoms of ovarian cysts are usually progressive abdominal distention, non-specific diffuse abdominal pain, vaginal bleeding, and symptoms related to organ compression, such as constipation, early satiety, vomiting, and frequent micturition [8]. Most giant ovarian cysts are treated by surgery. Surgical indications include a rapidly growing or symptomatic cyst, and when its malignant potential cannot be excluded [9].

Classification of ovarian cysts 

Functional ovarian cysts, cysts that develop as part of the menstrual cycle and are usually harmless and short-lived, are the most common type. Pathological ovarian cysts, cysts that form as a result of abnormal cell growth, are much less common. They can be benign or malignant. Benign pathological cysts are further divided into the following types: (1) dermoid cysts (also known as teratomas), (2) cystadenomas, (3) endometriomas (cysts related to endometriosis).

Malignant cysts most commonly include cystadenocarcinoma [10]. Ovarian serous cyst adenomas, benign tumors arising from the ovarian epithelium, are the most common type of giant ovarian cysts. Despite their rarity, clinicians may encounter such cases in their practice, and patients often present with vague complaints that can masquerade as other common conditions, such as ascites, distended bladder, hydronephrosis, ascites, accentuated obesity, fibroids, intra-abdominal and adnexal masses, and mesenteric cysts [1]. In the present case, the ovarian cyst masquerades as pregnancy, which is extremely rare. The patient attributed her non-specific symptoms to pregnancy, further delaying the diagnosis. The most serious implications of delayed diagnosis can be cyst torsion and cyst rupture. The aim of this study was to highlight large ovarian epithelial cysts that manifest unexpectedly, in order to reduce the risk of misdiagnosis, delayed diagnosis, and inadequate management.

## Case presentation

A 30-year-old woman, with a history of four previous pregnancies (G0, P4), presented to our center with a significantly distended abdomen, initially suggestive of pregnancy. She had not undergone any medical examination in the past. She reported cessation of her menstrual cycle eight months prior, accompanied by gradual abdominal enlargement. Initial symptoms, such as nausea, vomiting, and abdominal fullness, further reinforced her belief in pregnancy. She came from a low socioeconomic background in a rural setting with no medical facilities. Her previous pregnancies were uneventful. The pregnancies were diagnosed and managed at home without any medical intervention. There were similar symptoms of weakness, nausea, and vomiting in all her pregnancies. However, she recently developed dull abdominal pain, which was persistent, prompting her to seek medical attention at a primary health centre, from where she was referred to our center.

Upon examination, the patient exhibited pallor, dehydration, and diminished breath sounds at the pulmonary bases. Her abdomen was markedly distended, with stretch marks and edema of the abdominal wall, and was depressible on palpation with diffuse tenderness, but without guarding. Bowel sounds were diminished, and percussion produced a dull note. Notably, her limbs were thin. On speculum examination, the cervix was found to be hypertrophied and congested, while the vagina appeared healthy. However, per-vaginal examination revealed that the uterus was not palpable due to a cystic mass occupying the entire abdomen and pelvis.

**Figure 1:**
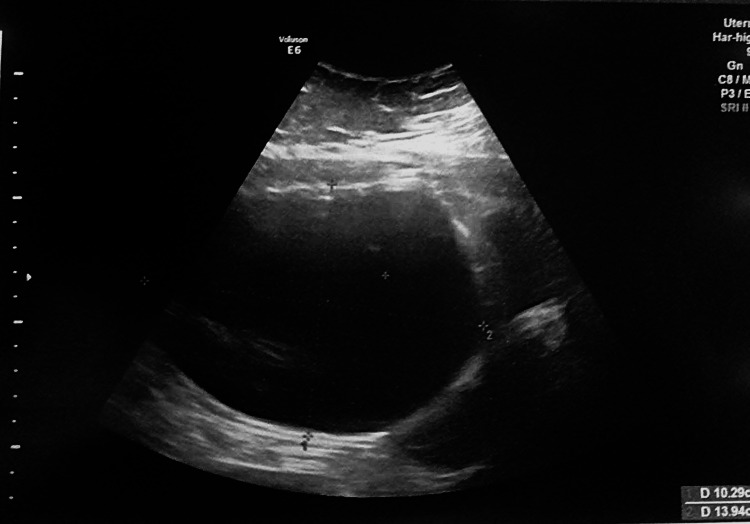
Ultrasound imaging showing a fluid filled massive cyst.

A urine human chorionic gonadotropin (hCG) test was done and found negative. Laboratory results revealed a hemoglobin level of 10 g/dL, while an abdominal ultrasound scan indicated a large, fluid-filled cyst originating from the right ovary (Figure 1). Tumor markers, including CA-125, CEA, and AFP, were within normal limits, supporting the diagnosis of a benign cyst. Additional hematological and biochemical serum tests yielded normal results. Hence, a provisional diagnosis of a giant multilocular ovarian cyst of right ovarian origin was made.

**Figure 2:**
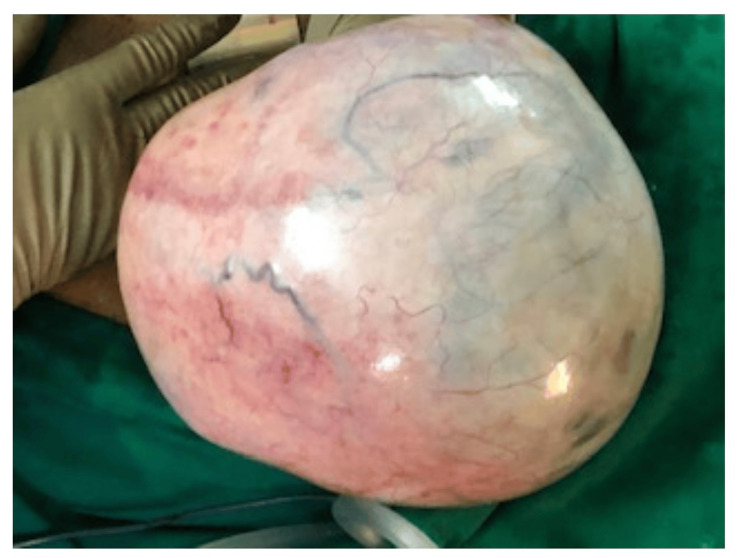
Giant ovarian cyst with intact membranes, measuring 54×51×26 cm.

Following thorough counseling and informed consent, the patient underwent a right ovarian cystectomy via a laparotomy. Laparotomy was done instead of laparoscopy because the ovarian cyst was extremely large. The massive size of the cyst made it technically challenging to manage laparoscopically, requiring a more direct and open approach via laparotomy to safely excise the cyst and ensure optimal patient care. The uterus, omentum, left ovary, and peritoneum appeared normal. A thorough examination of other intra-abdominal organs, like the liver and kidneys, was carried out successfully. The cyst was excised with membranes intact, and it measured 54×51×26 cm and weighed 10.9 kg (Figure 2).

A successful right ovarian cystectomy was performed. The left ovary was preserved effectively. This preservation was significant given the patient’s age (30 years) and the importance of maintaining ovarian function for hormonal and reproductive health. This surgical approach enabled effective removal of the cyst while ensuring optimal patient care. The patient had an uneventful postoperative recovery and was discharged on the fifth postoperative day.

## Discussion

Giant ovarian cysts (GOCs) remain a diagnostic challenge, particularly in resource-limited settings. Delayed presentation and surgical interventions are common, largely due to the non-specific nature of symptoms, which often only become apparent at an advanced stage. Detecting and diagnosing ovarian cysts early can be tough because patients often don't seek medical help until the cyst or tumor grows large enough to cause symptoms. Initial symptoms like abdominal pain or bloating can have many possible causes, making it hard to identify the root cause. When a patient first sees their primary care provider, it can be challenging to pinpoint what's causing the pain. Raising awareness about ovarian cysts is key to ensuring timely and effective diagnosis and treatment, which ultimately improves patient outcomes. In rural India, socioeconomic factors, cultural beliefs, and fear of surgery contribute to patients seeking medical attention only when symptoms become severe.

There are reported cases in literature of a giant ovarian cyst masquerading as a distended bladder, hydronephrosis, ascites, accentuated obesity, fibroids, intra-abdominal and adnexal masses, and a mesenteric cyst. However, a giant ovarian cyst masquerading as pregnancy is extremely rare and undocumented [1,2,7,11]. Abdominopelvic ultrasound scans can be a valuable diagnostic tool in these situations, enabling healthcare providers to make timely diagnoses and provide effective treatment.

Management of ovarian cysts depends on the patient’s age, menopausal status, the size and structure of the cyst, and the presence of complications. The management of giant ovarian cysts poses a significant challenge, particularly when the healthy ovary has to be preserved. In a 30-year-old female, preserving the ovaries is generally recommended because they play a crucial role in hormone production, reproductive health, and overall well-being. Ovaries produce estrogen, which helps maintain bone density, cardiovascular health, and sexual function. Preserving ovaries in a young woman can also avoid premature menopause and its associated symptoms and long-term health risks.

Complete surgical enucleation of the cyst is the gold standard approach for the management of a giant chylous cyst. Conservative surgical intervention, cystectomy with salpingo-oophorectomy, is adequate for young patients with benign ovarian lesions [11]. For giant ovarian cysts that seem benign after thorough evaluation, laparoscopic surgery with decompression is a viable option. To minimize risks, care must be taken to prevent spillage during decompression. However, caution is advised when malignancy can't be completely ruled out [12].

Due to the patient's extremely late presentation, which resulted in a massive ovarian cyst, a laparotomy was necessitated over laparoscopy, as the sheer size and complexity of the present case demanded an open surgical approach to ensure safe and effective management. A laparotomy had to be performed to avoid the risk of cyst rupture during laparoscopic removal of the giant ovarian cyst, particularly due to the limited working space and potential difficulties in maneuvering instruments within a large cyst.

## Conclusions

Very few cases of giant ovarian cysts that present masquerading as pregnancy have been reported. This case is reported to increase the suspicion index of giant ovarian cysts in women presenting with abdominal distention in rural communities with low socioeconomic conditions. The patient initially attributed her symptoms to pregnancy, further delaying her seeking medical attention. Limited access to diagnostic facilities, including ultrasonography, in primary healthcare settings further complicates timely diagnosis.

While laparoscopic surgery can be technically demanding in such cases, with risk of cyst rupture, it should be considered in young patients with benign ovarian cysts, as indicated by normal tumor markers and imaging modalities. In the present case, late presentation and massive cyst size made laparotomy mandatory. Hence, this particular case highlights the importance of timely diagnosis and appropriate management.
